# 伴t（4;14）初诊多发性骨髓瘤的临床特征及预后分析

**DOI:** 10.3760/cma.j.cn121090-20250618-00285

**Published:** 2026-03

**Authors:** 睿 郭, 欣 程, 旭星 沈, 媛媛 金, 丽娟 陈

**Affiliations:** 1 南京医科大学第一附属医院、江苏省人民医院血液科，南京 210029 Department of Hematology, The First Affiliated Hospital of Nanjing Medical University, Jiangsu Province Hospital, Nanjing 210029, China; 2 南京医科大学第一临床医学院，南京 211166 The First School of Clinical Medicine, Nanjing Medical University, Nanjing 211166, China

**Keywords:** 多发性骨髓瘤, t（4;14）, 细胞遗传学, 荧光原位杂交, Multiple myeloma, t（4;14）, Cytogenetics, Fluorescence in situ hybridization

## Abstract

**目的:**

分析伴t（4;14）初诊多发性骨髓瘤（NDMM）患者的临床特征及预后。

**方法:**

回顾性分析2017年4月至2024年10月在南京医科大学第一附属医院确诊的431例NDMM患者，采用胞质轻链免疫荧光结合荧光原位杂交（cIg-FISH）技术进行细胞遗传学检测，分析伴t（4;14）患者的临床特征及预后。

**结果:**

t（4;14）阳性组患者69例（16.0％），t（4;14）阴性组患者362例（84.0％），t（4;14）阳性组IgG型、血小板减少、合并1q21获得/扩增的比例较t（4;14）阴性组高（*P*值均<0.05）。t（4;14）阳性组无进展生存（PFS）期显著短于阴性组（27个月对39个月，*P*＝0.014），但中位总生存（OS）期的差异无统计学意义（未达到对未达到，*P*＝0.056）。69例t（4;14）阳性患者中单纯t（4;14）阳性组18例，合并高危细胞遗传学异常（HRCA）组51例［50例合并1q21获得/扩增或del（1p32）］，两组患者的中位PFS期分别为33（95％ *CI*：24～42）个月和26（95％ *CI*：16～41）个月，中位OS期均未达到。t（4;14）合并HRCA组的PFS和OS较t（4;14）阴性组差（*P*值分别为0.030、0.026）。t（4;14）阳性患者中25例进行了早期自体造血干细胞移植，中位PFS期较未移植患者有延长趋势［41个月（95％ *CI*：14个月～未达到）对26（95％ *CI*：18～35）个月］，但差异无统计学意义（*P*＝0.135）；22例患者进行了完整的疗效评估，移植前和移植后3个月≥完全缓解率由40.9％提升至63.6％，≥非常好的部分缓解率由81.8％提升至90.9％。Cox回归分析显示，校正治疗方案差异后，t（4;14）仍是影响NDMM患者PFS的独立危险因素（*HR*＝1.608，95％*CI*：1.103～2.346，*P*＝0.014），而非OS的独立危险因素。

**结论:**

在新药时代，t（4;14）是影响NDMM患者PFS的独立危险因素，但对OS的影响不显著，t（4;14）合并HRCA患者预后更差。

## Abstract (en)

**Objective:**

This study aimed to analyze the clinical characteristics and prognosis of patients with newly diagnosed multiple myeloma（NDMM）with t（4;14）.

**Methods:**

Data of 431 patients with NDMM diagnosed in the First Affiliated Hospital of Nanjing Medical University between April 2017 and October 2024 were retrospectively analyzed. Cytoplasmic light chain immunofluorescence with fluorescence in situ hybridization was used for cytogenetic testing to analyze the clinical characteristics and prognosis of patients with t（4;14）.

**Results:**

Among the enrolled patients, 69（16.0％）were positive for t（4;14）, and 362（84.0％）were negative. Compared with the t（4;14）-negative group, patients in the t（4;14）-positive group demonstrated significantly higher proportions of IgG subtype, thrombocytopenia, and concomitant gain/amplification of chromosome 1q21（all *P*<0.05）. The median progression-free survival（PFS）of patients with t（4;14）was significantly shorter than that of patients with negative t（4;14）（27 months *vs* 39 months, *P*＝0.014）. However, no statistically significant difference in median overall survival（OS）was observed between the two groups（not reached *vs* not reached, *P*＝0.056）. Among the 69 patients with t（4;14）, 18 had isolated t（4;14）and 51 had concomitant high-risk cytogenetic abnormalities（HRCA）, including 50 patients with 1q21 gain/amplification or 1p32 deletion. The median PFS was 33（95％ *CI*: 24–42）months in patients with isolated t（4;14）and 26（95％ *CI*: 16–41）months in those with t（4;14）plus HRCA, whereas the median OS was not reached in either group. Patients with t（4;14）combined with HRCA demonstrated inferior PFS and OS compared with the t（4;14）-negative group（*P*＝0.030 and *P*＝0.026, respectively）. Among patients with t（4;14）, 25 underwent early autologous hematopoietic stem cell transplantation and demonstrated a trend toward longer median PFS compared with those who did not undergo transplantation［41（95％ *CI*: 14– not reached）months *vs* 26（95％ *CI*: 18–35）months］, although the difference was not statistically significant（*P*＝0.135）. Treatment response was assessed in 22 transplanted patients, revealing that the rate of ≥ complete response increased from 40.9％ before transplantation to 63.6％ at 3 months post-transplantation, and the rate of ≥ very good partial response increased from 81.8％ to 90.9％. Multivariate Cox regression analysis after adjustment for treatment-related factors identified that t（4;14）remained an independent adverse prognostic factor for PFS in patients with NDMM（*HR*＝1.608, 95％ *CI*: 1.103–2.346, *P*＝0.014）, but not for OS.

**Conclusion:**

In the era of novel agents, t（4;14）was identified as an independent risk factor for PFS in patients with NDMM, although it did not significantly affect OS. Patients with t（4;14）combined with other HRCA demonstrated a worse prognosis.

多发性骨髓瘤（MM）目前仍是一种不可治愈的恶性浆细胞增殖性疾病，生存期存在高度异质性，高危细胞遗传学异常（HRCA）是影响预后的重要因素，但随着靶向治疗、免疫治疗和造血干细胞移植等的应用，高危细胞遗传学的概念在不断变化。t（4;14）在多个危险分层系统［包括修订的国际分期系统（R-ISS）、国际骨髓瘤工作组（IMWG）危险分层和梅奥诊所骨髓瘤分层和风险适应治疗（mSMART）3.0系统］均被列为HRCA，但2024年IMWG及mSMART 4.0对高危的概念又有了新的诠释。本研究对伴t（4;14）的初诊MM（NDMM）患者的临床特征进行回顾性分析，以了解t（4;14）对疗效和生存的影响。

## 病例与方法

1. 病例：本研究纳入南京医科大学第一附属医院自2017年4月至2024年10月诊断的431例NDMM患者，对NDMM患者的临床特征［年龄、性别、分型、DS分期、ISS分期、R-ISS分期、R2-ISS分期、髓外病变、HGB、血清肌酐、血清LDH、PLT和细胞遗传学异常（CA）等］进行回顾性分析。MM的诊断标准符合《中国多发性骨髓瘤诊治指南（2024年修订）》[1]。本研究经江苏省人民医院医学研究伦理委员会批准（批号：2022-SR-448）。

2. 胞质轻链免疫荧光结合荧光原位杂交（cytoplasmic light chain immunofluorescence with fluorescence in situ hybridization，cIg-FISH）技术检测染色体核型异常：釆集NDMM患者骨髓液经密度梯度离心法分离单个核细胞，细胞离心机（购自美国艾普迪公司）离心5 min（离心半径10 cm，1 000 r/min）涂片，干燥后无水乙醇固定，37 °C孵箱老化，90％甲酰胺预变性，−20 °C预冷的梯度乙醇进行脱水、干燥后加探针，封固后杂交，杂交温度为75 °C 15 min、42 °C大于15 h。次日，50％甲酰胺（45 °C水浴）和2×氯化钠柠檬酸钠缓冲液（37 °C水浴）洗去非特异性信号，Kappa或Lambda抗体染胞质轻链，加抗淬灭剂封固，用荧光显微镜（购自德国蔡司公司）进行原始荧光图像的采集，计数100个标记细胞进行分析，计算阳性信号的百分比[2]。

cIg-FISH的探针购自美国雅培公司，主要包括CKS1B/CDKN2C、TP53/CEP17、IGH/FGFR3和IGH/MAF，分别定位染色体1q21.2/1p32.3、17p13.1/17p11.1-q11.1、t（4;14）（p16.3;q32.3）、t（14;16）（q32.3;q23）。阳性阈值采用欧洲骨髓瘤工作组（European Myeloma Network，EMN）标准，CKS1B/CDKN2C、TP53/CEP17的阳性标准大于20％，IGH/FGFR3和IGH/MAF的阳性标准大于10％[3]，文中将1q21获得/扩增定义为1q21+。

3. 治疗方案与随访：431例患者的诱导化疗方案包括以硼替佐米为主的蛋白酶体抑制剂（PI）、以来那度胺为主的免疫调节剂（IMiD）和PI联合IMiD，部分患者在治疗过程中接受了达雷妥尤单抗（daratumumab，Dara），在诱导化疗后部分患者进行了自体造血干细胞移植（auto-HSCT）。采用电话或查阅病历的方式进行随访，随访截止时间为2025年2月28日。

4. 疗效评价：采用IMWG的疗效标准评价患者的疗效[4]，分为严格意义的完全缓解（sCR）、完全缓解（CR）、非常好的部分缓解（VGPR）、部分缓解（PR）和疾病进展（PD）。总有效率（ORR）为sCR率+CR率+VGPR率+PR率，无进展生存（PFS）期为患者自接受一线诱导治疗至疾病发生进展的时间，总生存（OS）期为患者自确诊到死亡或末次随访所经历的时间。

5. 统计学处理：采用SPSS 27.0和Graphpad Prism 10.0软件进行统计学分析。定性资料用例数（百分比）表示，定量资料用*M*（范围）表示，定性资料的比较用*χ*^2^检验。采用Kaplan-Meier法绘制患者的生存曲线，组间比较采用Log-rank检验，预后影响因素的分析采用Cox比例风险回归模型。全部统计学分析采用双侧检验，*P*<0.05为差异有统计学意义。

## 结果

1. 基线资料：对431例NDMM患者的临床数据进行分析，其中男245例（56.8％），女186例（43.2％），中位年龄为63（29～86）岁。按照是否伴t（4;14）将患者分为两组，t（4;14）阳性组患者69例（16.0％），t（4;14）阴性组患者362例（84.0％），其基线数据详见表1，两组患者性别、年龄、DS分期、ISS分期、血清肌酐、HGB、LDH、骨髓原始和幼稚浆细胞比例的差异均无统计学意义（*P*值均>0.05），分型和PLT的差异均有统计学意义（*P*值均<0.05），t（4;14）阳性组患者IgG型和PLT减少的比例高于t（4;14）阴性组（表1）。

**表1:** t（4;14）阴性与阳性组多发性骨髓瘤患者基线临床特征比较［例（％）］

临床特征	t（4;14）阴性组 （362例）	t（4;14）阳性组 （69例）	*χ*^2^值	*P*值
性别			0.222	0.637
男	204（56.4）	41（59.4）		
女	158（43.6）	28（40.6）		
年龄			2.400	0.121
≤65岁	205（56.6）	46（66.7）		
>65岁	157（43.4）	23（33.3）		
分型			28.700	<0.001
IgG	149（41.2）	48（69.6）		
IgA	82（22.7）	17（24.6）		
IgD	21（5.8）	0（0）		
轻链型	96（26.5）	2（2.9）		
其他	14（3.9）	2（2.9）		
DS分期			4.481	0.106
Ⅰ期	21（5.8）	0（0）		
Ⅱ期	58（16.0）	10（14.5）		
Ⅲ期	283（78.2）	59（85.5）		
ISS分期			4.006	0.135
Ⅰ期	67（18.5）	6（8.7）		
Ⅱ期	135（37.3）	28（40.6）		
Ⅲ期	160（44.2）	35（50.7）		
遗传学异常				
1q21+	189（52.2）	48（69.6）	7.053	0.008
del（1p32）	34（9.4）	4（5.8）	0.932	0.334
del（17p13）	32（8.8）	8（11.6）	0.522	0.470
t（14;16）	3（0.8）	0（0）	<0.001	1.000
伴髓外病变	79（21.8）	15（21.7）	<0.001	0.988
血清肌酐			1.429	0.232
≤177 µmol/L	281（77.6）	58（84.1）		
>177 µmol/L	81（22.4）	11（15.9）		
HGB			1.417	0.234
<100 g/L	258（71.3）	54（78.3）		
≥100 g/L	104（28.7）	15（21.7）		
乳酸脱氢酶			2.445	0.118
≤220 U/L	261（72.1）	56（81.2）		
>220 U/L	101（27.9）	13（18.8）		
PLT			8.111	0.004
<125×10^9^/L	87（24.0）	28（40.6）		
≥125×10^9^/L	275（76.0）	41（59.4）		
骨髓原始和幼稚浆细胞比例≥60％	32（8.8）	10（14.5）	2.106	0.147
诱导治疗方案			1.751	0.417
PI为主	135（37.3）	27（39.1）		
IMiD为主	26（7.2）	2（2.9）		
PI+IMiD	201（55.5）	40（58.0）		

**注** HGB：血红蛋白；PLT：血小板计数；PI：蛋白酶体抑制剂；IMiD：免疫调节剂

进一步对t（4;14）阳性患者是否合并其他HRCA进行分析。单纯t（4;14）阳性患者18例（26.1％）。在伴有其他HRCA的病例中，以合并单纯1q21+最为常见［40例（58.0％）］，t（4;14）合并1q21+及del（17p13）6例（8.7％），合并1q21+及del（1p32）2例（2.9％）。此外，合并单纯del（1p32）、单纯del（17p13）及同时合并del（1p32）与del（17p13）各1例（1.4％）。t（4;14）阳性患者较t（4;14）阴性患者更易合并1q21+（69.6％对52.2％，*P*＝0.008）。

2. 治疗疗效分析：诱导治疗4个周期后进行疗效评估，发现t（4;14）阴性组患者的ORR、sCR率、CR率、VGPR率、PR率分别为95.9％、28.7％、12.2％、32.6％和22.4％，t（4;14）阳性组患者的ORR、sCR率、CR率、VGPR率、PR率分别为92.7％、18.8％、10.2％、42.0％和21.7％。t（4;14）阴性组和阳性组患者ORR、≥VGPR率和≥CR率的差异均无统计学意义（*P*值分别为0.262、0.063、0.672）。

进一步对t（4;14）阳性NDMM患者的治疗方案进行分析，27例患者应用以PI为主的方案诱导治疗，ORR为88.9％（24/27）；40例患者应用PI联合IMiD方案治疗，ORR为97.5％（39/40）［其中26例患者应用PI联合IMiD但未应用Dara，ORR为96.2％（25/26）；14例患者应用PI联合IMiD和Dara，ORR为100.0％（14/14）］；仅2例患者应用以IMiD为主的方案治疗，未单独纳入分析。40例应用PI联合IMiD的患者中，未应用和应用Dara患者PFS和OS的差异均无统计学意义（*P*值分别为0.214、0.505）。69例t（4;14）阳性NDMM患者中，25例（36.2％）进行了早期auto-HSCT，移植患者的中位PFS期较未移植患者有延长趋势［41个月（95％ *CI*：14个月～未达到）对26（95％ *CI*：18～35）个月］，但差异无统计学意义（*P*＝0.135）；移植组与未移植组OS的差异虽无统计学意义（均未达到，*P*＝0.320），但移植组的5年OS率较未移植组有所提高（65.9％对50.7％）。移植组中22例患者进行了完整的疗效评估，结果显示，移植后3个月≥CR率由移植前的40.9％提高至63.6％，≥VGPR率由移植前的81.8％提高至90.9％，具体疗效评价见表2。

**表2:** 431例初诊多发性骨髓瘤患者的疗效评价情况

疗效	所有患者		t（4;14）阳性患者			t（4;14）阳性行auto-HSCT患者	
	t（4;14）阳性（69例）	t（4;14）阴性（362例）	PI为主（27例）	PI+IMiD（未使用Dara）（26例）	PI+IMiD+Dara（14例）	移植前（22例）	移植后3个月（22例）
sCR	13（18.8）	104（28.7）	5（18.5）	5（19.2）	3（21.4）	7（31.8）	11（50.0）
CR	7（10.2）	44（12.2）	2（7.4）	5（19.2）	0（0）	2（9.1）	3（13.6）
VGPR	29（42.0）	118（32.6）	12（44.4）	11（42.3）	6（42.9）	9（40.9）	6（27.3）
PR	15（21.7）	81（22.4）	5（18.5）	4（15.4）	5（35.7）	4（18.2）	2（9.1）
PD	5（7.3）	15（4.1）	3（11.1）	1（3.8）	0（0）	0（0）	0（0）

**注** sCR：严格意义的完全缓解；CR：完全缓解；VGPR：非常好的部分缓解；PR：部分缓解；PD：疾病进展；PI：蛋白酶体抑制剂；IMiD：免疫调节剂；Dara：达雷妥尤单抗；auto-HSCT：自体造血干细胞移植

3. 生存分析：截至2025年2月28日，患者中位随访38（3～92）个月，431例患者中345例存活，86例死亡，174例PD，中位PFS期为39（95％*CI*：34～44）个月，中位OS期未达到，5年OS率为68.2％。t（4;14）阳性组和t（4;14）阴性组的中位PFS期分别为27（95％ *CI*：23～41）个月和39（95％ *CI*：34～51）个月，差异有统计学意义（*P*＝0.014），OS期的差异无统计学意义（均未达到，*P*＝0.056）（图1）。

**图1:**
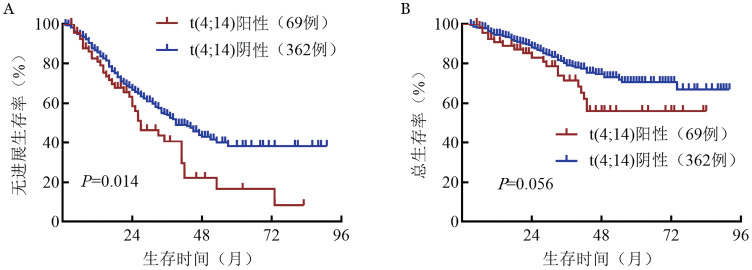
t（4;14）阳性与阴性多发性骨髓瘤患者的无进展生存（A）和总生存（B）曲线

根据t（4;14）阳性NDMM患者是否合并其他HRCA（参考mSMART 3.0标准）将患者分为t（4;14）阴性组、单纯t（4;14）阳性组和t（4;14）阳性合并HRCA组，三组的中位PFS期分别为39（95％ *CI*：34～51）个月、33（95％ *CI*：24～42）个月和26（95％ *CI*：16～41）个月，三组患者的中位OS期均未达到，5年OS率分别为70.7％、65.7％和51.2％（图2）。单纯t（4;14）阳性组与阴性组PFS期和OS期的差异均无统计学意义（中位PFS期：33个月对39个月，中位OS期均未达到，*P*值均>0.05），t（4;14）阳性合并HRCA组的PFS期和OS期均较t（4;14）阴性组明显缩短（中位PFS期：26个月对39个月，中位OS期均未达到，*P*值分别为0.030、0.026）。

**图2:**
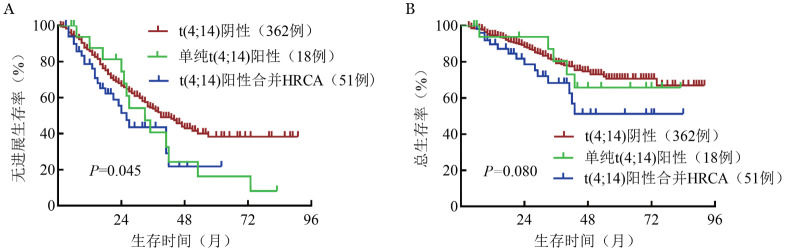
t（4;14）阴性组、单纯t（4;14）阳性组和t（4;14）阳性合并高危细胞遗传学异常（HRCA）组的无进展生存（A）和总生存（B）曲线

单因素和多因素分析显示，PLT≤125×10^9^/L、LDH≥220 U/L、初诊时伴髓外病变、使用Dara、auto-HSCT、伴del（1p32）和伴t（4;14）均为影响PFS的独立危险因素（*P*值均<0.05），其中t（4;14）的*HR*为1.608，在各类HRCA中仅次于del（1p32）；LDH≥220 U/L、auto-HSCT、del（1p32）和del（17p13）均为影响OS的独立危险因素（*P*值均<0.05），t（4;14）对OS的影响不显著（表3）。

**表3:** 初诊多发性骨髓瘤患者无进展生存和总生存影响因素的单因素与多因素分析

因素	无进展生存				总生存			
	单因素分析		多因素分析		单因素分析		多因素分析	
	*HR*（95％*CI*）	*P*值	*HR*（95％*CI*）	*P*值	*HR*（95％*CI*）	*P*值	*HR*（95％*CI*）	*P*值
骨髓原始和幼稚浆细胞比例≥60％	1.546（0.988～2.419）	0.056			1.988（1.121～3.525）	0.019	1.568（0.868～2.834）	0.136
HGB<100 g/L	0.769（0.543～1.090）	0.140			0.950（0.589～1.532）	0.832		
肌酐≥177 µmol/L	1.510（1.071～2.129）	0.019	1.259（0.862～1.838）	0.233	2.096（1.334～3.295）	0.001	1.446（0.892～2.342）	0.134
PLT≤125×10^9^/L	1.620（1.176～2.231）	0.003	1.536（1.100～2.145）	0.012	1.399（0.886～2.209）	0.149		
LDH≥220 U/L	1.791（1.314～2.441）	<0.001	1.412（1.013～1.968）	0.042	2.163（1.406～3.327）	<0.001	1.842（1.179～2.879）	0.007
初诊时伴髓外病变	1.620（1.170～2.242）	0.004	1.632（1.151～2.314）	0.006	1.420（0.887～2.276）	0.144		
诱导治疗方案	0.813（0.697～0.949）	0.009	0.900（0.765～1.059）	0.205	0.803（0.644～1.002）	0.052		
使用Dara	1.716（1.245～2.366）	<0.001	1.530（1.100～2.129）	0.011	0.678（0.394～1.168）	0.162		
auto-HSCT	0.434（0.309～0.609）	<0.001	0.474（0.334～0.672）	<0.001	0.346（0.204～0.589）	<0.001	0.379（0.218～0.657）	<0.001
del（1p32）	2.588（1.663～4.025）	<0.001	2.136（1.332～3.427）	0.002	4.318（2.493～7.480）	<0.001	2.693（1.478～4.906）	0.001
1q21+	1.320（0.978～1.783）	0.070			1.303（0.848～2.001）	0.227		
del（17p13）	1.426（0.903～2.251）	0.128			2.053（1.138～3.705）	0.017	2.004（1.087～3.694）	0.026
t（4;14）	1.567（1.090～2.255）	0.015	1.608（1.103～2.346）	0.014	1.618（0.981～2.669）	0.059		

**注** PLT：血小板计数；LDH：乳酸脱氢酶；Dara：达雷妥尤单抗；auto-HSCT：自体造血干细胞移植

## 讨论

MM是一种起源于单克隆浆细胞的恶性血液病，以贫血、高钙血症、肾功能不全和骨性病变为主要特征[5]，MM的异质性较高，精确分层有助于实现患者的个体化治疗。在诸多MM预后不良的预测因素中，CA被广泛应用，美国国家综合癌症网络和美国梅奥医学中心把del（1p32）、1q21+、del（17p13）、t（4;14）、t（14;16）、t（14;20）等CA列为HRCA，但近期也有研究认为t（4;14）仅提示中危[6]，甚至与患者的PFS和OS无关[7]–[8]。

本研究中，t（4;14）阳性率为16.0％，与文献报道基本一致[9]–[10]，t（4;14）阳性患者PLT减少的比例增高，可能与疾病进展导致的血小板过度消耗相关[11]。D'Agostino等[12]的研究显示，ISS分期Ⅱ期/Ⅲ期、LDH升高、1q21+、del（17p13）和t（4;14）可影响NDMM患者的PFS和OS，于2022年提出R2-ISS分期，其中t（4;14）被赋予1分。然而在本次研究中，虽然t（4;14）阳性NDMM的PFS期较t（4;14）阴性患者显著缩短，但OS的差异无统计学意义，Cox回归分析也显示，t（4;14）仅是影响PFS的独立危险因素。Rajkumar等[13]的研究结果显示，t（4;14）仅可独立预测早期复发风险，与本研究结果一致。

Stong等[14]的研究显示，仅有30％～40％的t（4;14）阳性NDMM患者体现临床高危，考虑该部分患者可能同时合并其他HRCA，本研究73.9％（51/69）伴t（4;14）的NDMM患者合并其他HRCA，这个数据可能与中国MM患者1q21+阳性比例较高有关。本研究发现，t（4;14）阳性患者更易合并1q21+，比例高达94.1％，据相关文献报道，NDMM双打击患者中最常见的组合就是t（4;14）与1q21+[15]，这些双打击MM患者常伴高侵袭性、高早期死亡率和更差的预后，属于“超高风险”亚组，可能是由于其他HRCA增强了t（4;14）的不良预后作用[16]。IMWG[17]和mSMART 4.0均认为t（4;14）仅在同时合并1q21+或del（1p32）的情况下才是高风险特征。本研究中，单纯t（4;14）阳性组与合并HRCA组PFS和OS的差异均无统计学意义，可能与单纯t（4;14）组例数较少及治疗方案不统一有关。但研究发现，和单纯t（4;14）阳性组相比，合并HRCA组［其中50例合并1q21+或del（1p32）］的中位PFS期由33个月缩短至26个月，5年OS率由65.7％下降至51.2％；且t（4;14）合并HRCA组患者的PFS期和OS期均较t（4;14）阴性组显著缩短，进一步证明该类患者预后较差。

既往研究显示，PI联合IMiD方案，尤其是VRD方案，可显著改善NDMM患者的PFS与OS[18]。本研究进一步显示，在t（4;14）阳性患者中，PI联合IMiD方案的ORR为97.5％，高于以PI为基础的方案（88.9％），提示其初始减瘤能力更强，临床上宜优先采用以PI+IMiD为核心的诱导治疗。尽管治疗方案加入Dara的14例患者的ORR达100％，但PFS和OS均未见显著获益，提示在VRD方案的基础上叠加Dara未必能延长生存期，受样本量较小所限，上述结论仍待更大规模研究验证。

一项多中心、随机、开放标签的Ⅲ期研究显示，与VMP方案（硼替佐米+美法仑+泼尼松）强化治疗相比，auto-HSCT组患者PFS期显著延长，其中包含11％的t（4;14）阳性患者[19]。本研究t（4;14）阳性患者auto-HSCT组与非auto-HSCT组的生存差异虽无统计学意义，但auto-HSCT组的中位PFS期呈延长趋势，且移植后3个月的≥CR率与≥VGPR率较移植前分别提高22.7％与9.1％。提示在新药时代，auto-HSCT对于符合条件的t（4;14）阳性NDMM患者仍具临床价值，可作为巩固治疗的重要组成部分。在校正治疗方案差异后，t（4;14）仍是影响PFS的独立不良因素，其不良预后意义在新药时代依然显著，合并其他HRCA时，基于PI的新药方案仍难以完全改善疗效。随着免疫治疗的推进，前沿研究已为t（4;14）阳性NDMM揭示多种潜在有效治疗靶点[20]–[23]，今后有望提升该类患者的缓解深度并延长生存时间。

综上所述，伴t（4;14）的NDMM患者具有独特的临床特征和预后，该类患者常合并其他HRCA，进一步增加了疾病的复杂性与危险性。基于PI及IMiD的诱导治疗方案虽能在一定程度上改善这类患者的疗效，但仍存在局限性。鉴于本研究为单中心回顾性研究，在样本量和随访时间上存在不足，未来期待通过多中心、大样本量数据进一步验证，为临床医师的治疗决策提供支持。
